# Supplementary material for: Challenges and Opportunities for the Translation of Single-Cell RNA Sequencing Technologies to Dermatology
Source: Life (Basel). 2022 Jan 4;12(1):67. doi: 10.3390/life12010067 (PMC8781146; doi:10.3390/life12010067)
Supplement: Supplementary file 1 [file life-12-00067-s001.zip › life-1512212-supplementary.pdf]

Table S1. Presence of mouse fibroblast subpopulation markers in human fibroblast subpopulations.

**Col15a1**	ALL		Anxa3	-		Ccl21a	-		Clca3a1	-		Ncam1	-
Gsn	A1/B		Ly6c1	-		Clu	A/B2		Tinagl1	-		Sfrp1	C
Abca8a	A1/B/C2		Ly6a	-		**Ccl19**	B2		Igfbp7	B		Tgm2	-
Smoc2	A		Cd248	A1		Apoe	B		B2m	ALL		Bmp4	A2/C
Fbln2	A1/A4		**Pi16**	A1/C1		Des	-		Ly6e	A2		Nkx2-3	-
Dcn	A1/C4		Igfbp6	A1/C4		Enpp2	ALL		Ifitm3	B2		Spon2	D
Lum	A1/C4		Timp2	A		C3	B2		H2-D1	-		**Fbln1**	A
Entpd2	C4		Emilin2	A1		Csf2rb	-		Lhfp	-		Plac8	-
Sparcl1	C2		Dpp4	A1/A3		Srgn	-		Emilin1	ALL		Rps12	ALL
Htra3	A4		Ackr3	A		Vtn	-		**Coch**	C2		Mfap4	ALL
Cygb	B		Lrrn4cl	A		B2m	ALL		Slc40a1	C2		Tmem176a	B
Ccdc80	A1/B		Sema3c	A1/A3		Tmem176b	B		Plvap	-		Hes1	B
Penk	-		Anxa1	ALL		Cpxm1	A2/C1		Ifit3	-		Pltp	A/C1
Crispld2	C4		Clec3b	A1		Mfge8	C1		Sept4	-		Ltbp4	C2/C4
Dpep1	C1		Fn1	A1/A4/C4		Tmem176a	B2		Gdpd2	-		Fam198b	-
Anxa5	ALL		Ugdh	A1/C4		H2-D1	-		Tcf21	-		Tmem176b	B
Col4a1	A2/C1		Efhd1	-		Ifitm1	B2		Rtp4	-		Fhl1	A
Lama2	A2/C1		Metrnl	-		Cxcl12	B		Ifitm1	B		Ifitm1	B
Tcf4	A2/C2/C3		Fbn1	A1/A4		Bst1	-		Iigp1	-		Aldh1a3	-
Hspg2	ALL		Pla1a	-		C2	-		Frzb	-		Matn2	C1/C4
Hp	-		Inmt	C4		**Comp**	A2		**Hhip**	-		Bmp5	-
Esm1	-		Limch1	C2		1500015O10Rik	-		Mustn1	-		Tmem158	-
**Cxcl12**	B		**Npnt**	-		Fxyd6	B2		Grem2	A		Adamdec1	B2
Adipoq	-		Macf1	-		Cilp	A1/A4		Enpp2	A2/B2/C		Nbl1	A
Spp1	-		Mfap4	ALL		Ptn	B/C3		Rgs2	A2		**Bmp4**	-
Gdpd2	-		Csrp1	C4		Pdgfrl	A1		Aspn	C		Ednrb	C1
Cxcl14	A		Gyg	-		Ctgf	C1/A2		6330403K07Rik	-		Tmem119	A2/C1

Table S1. Ascensión et al. (continued)

Igfbp4	A		Fhl1	A		Cthrc1	A1		Sgcg	A		Aldh1a1	A1/B1
Gas6	A		Mettl7a1	-		Cst3	ALL		Cyp2e1	-		Nkx2-3	-
Runx1	A2/B1		Mgp	A1/B1		Timp1	ALL		Btg2	C2/C4		Tspan13	-
Ibsp	-		Cdo1	-		Vim	ALL		Thbs1	A2/C1		Emid1	C2
Kitl	-		Ppp1r14a	C		Plod2	-		P2ry14	B2/C4		Cald1	C1
Rgcc	A2		Sept4	-		Tm4sf1	C4		Sftpc	-		Rgs10	-
Pappa	-		Sod3	ALL		Prg4	A4		Acta2	C1		Fhl1	ALL
Serpine2	A2/ALL		Fmo2	C4		Ddah1	-		Pde4b	-		Tgm2	-
Kng2	-		Lbh	A2		Fibin	C2		Bmp5	-		Fhl2	A1
Cebpa	-		Pcolce2	A1/A4		Thbs4	A3/C2		Mgp	B		Sept4	-
Slc6a6	-		Itga8	-		Runx1	A2/B1		Cdh4	-		Tpm2	A1
1500009L16Rik	-		Hsd11b1	-		Fmod	C2		Hmgb1	ALL		Bmp7	-
Ebf3	-		Selenbp1	-		Col1a1	ALL		Tagln	C1/C4		Myl9	C1
